# Electrophysiology lab efficiency comparison between cryoballoon and point-by-point radiofrequency ablation: a German sub-analysis of the FREEZE Cohort study

**DOI:** 10.1186/s12872-022-03015-8

**Published:** 2023-01-09

**Authors:** Andreas Metzner, Florian Straube, Roland R. Tilz, Malte Kuniss, Georg Noelker, Juergen Tebbenjohanns, Dietrich Andresen, Heinrich Wieneke, Christoph Stellbrink, Jennifer Franke, Uwe Dorwarth, Phuong Lien Carion, Reece Holbrook, Matthias Hochadel, Jochen Senges, Ellen Hoffmann, Karl-Heinz Kuck, for the FREEZE Cohort Study Investigators, L. Q. Wu, A. Garcia-Alberola, T. Massa, G. Sabin, A. Franke, J. J. Souza, A. Stanley, S. G. Spitzer, S. Willems, T. Dierk, K. R. J. Chun, R. Borchard, K. H. Seidl, R. Zahn, G. Groschup, I. W. P. Obel, J. Brachmann, J. H. Gerds-Li, R. R. Gopal, J. Schrickel, T. Lewalter, A. Stanley, W. Moshage, L. Eckardt, W. Jung, P. Kremer, A. Lubinski, B. Schumacher, L. Lickfett, T. Münzel, C. Steinwender, M. Efremidis, T. Deneke, D. Q. Nguyen

**Affiliations:** 1 Department of Cardiology, Asklepios Klinik St. Georg, Hamburg, Germany; 2 Department of Cardiology, Universitätsklinikum Hamburg-Eppendorf, Martinistraße 52, Gebäude Ost 70, 20246 Hamburg, Germany; 3 Department of Cardiology and Internal Intensive Care Medicine, Heart Center Munich-Bogenhausen - Munich Municipal Hospital Group, Munich, Germany; 4 Department of Cardiology, Angiology, and Intensive Care Medicine, University Hospital Schleswig-Holstein, University Heart Centre Luebeck, Lübeck, Germany; 5 Department of Cardiology, Kerckhoff-Klinik, Bad Nauheim, Germany; 6 Herz- Und Diabeteszentrum Nordrhein-Westfalen, Bad Oeynhausen, Germany; 7 HELIOS Klinikum Hildesheim, Medizinische Klinik I – Kardiologie, Hildesheim, Germany; 8 Department of Cardiology Paul Gerhardt Diakonie gAG, Evangelisches Krankenhaus Hubertus, Berlin, Germany; 9 Klinik Für Kardiologie und Angiologie, Contilia Herz- Und Gefäßzentrum, Essen, Germany; 10 Department of Cardiology, Klinikum Bielefeld, Bielefeld, Germany; 11 CardioVascular Center Frankfurt, Frankfurt, Germany; 12 Medtronic International Trading Sàrl, Tolochenaz, Switzerland; 13 Medtronic, Inc., Mounds View, MN USA; 14 Stiftung Institut Fur Herzinfarktforschung, Ludwigshafen, Germany

**Keywords:** Atrial fibrillation, Ablation, Cryoablation, Radiofrequency ablation, Efficiency, Electrophysiology lab

## Abstract

**Background:**

Pulmonary vein isolation (PVI) is recommended to treat paroxysmal and persistent atrial fibrillation (AF). This analysis aimed to assess the hospital efficiency of single-shot cryoballoon ablation (CBA) and point-by-point radiofrequency ablation (RFA).

**Methods:**

The discrete event simulation used PVI procedure times from the FREEZE Cohort study to establish the electrophysiology (EP) lab occupancy time. 1000 EP lab days were simulated according to an illustrative German hospital, including 3 PVI cases per day using CBA at one site and RFA at the other.

**Results:**

The analysis included 1560 CBA patients and 1344 RFA patients from the FREEZE Cohort. Some baseline patients’ characteristics were different between groups (age, AF type, and some concomitant diseases), without being statistically associated to ablation procedure time. Mean procedure time was 122.2 ± 39.4 min for CBA and 160.3 ± 53.5 min for RFA (*p* < 0.0001). RFA was associated with a more than five-fold increase of cumulative overtime compared to CBA over the simulated period (1285 h with RFA and 253 h with CBA). 70.7% of RFA lab days included overtime versus 25.7% for CBA. CBA was associated with more days with an additional hour at the end of the EP lab shift compared to RFA (47.8% vs 11.5% days with one hour left, respectively).

**Conclusion:**

CBA is faster and more predictable than point-by-point RFA, and enables improvements in EP lab efficiency, including: fewer cumulative overtime hours, more days where overtime is avoided and more days with remaining time for the staff or for any EP lab usage.

*Clinical trial registration* NCT01360008 (first registration 25/05/2011).

**Supplementary Information:**

The online version contains supplementary material available at 10.1186/s12872-022-03015-8.

## Background

Atrial fibrillation (AF) is the most common sustained cardiac arrhythmia and is an increasingly pressing issue for health care systems in Western Europe countries due to the ageing population. Currently, at least 8 million inhabitants of the European Union suffer from AF, and the revised lifetime risk for AF in individuals over 55 years old increased from 1 out of 4 to 1 out of 3 [1].

According to the European Society of Cardiology guidelines, AF catheter ablation is a well-established treatment for the prevention of AF recurrences and is recommended to improve AF symptoms in patients with paroxysmal or persistent AF [1]. The cornerstone of AF catheter ablation is the complete pulmonary vein isolation (PVI), and can be accomplished with radiofrequency point-by-point circumferential lesions or single-shot ablation devices like the cryoballoon [2, 3]. In Germany, an estimated number of 92,220 ablations were performed in 2018, an increase of more than 5% within one year, according to a yearly survey among several hundred electrophysiology units [4]. Out of 237 responding ablation centres, 27% of all facilities performed up to 100 ablations per year, and 17.6% more than 500. The increasing number of patients with AF is a challenge for physicians, hospitals and the healthcare system in Germany. Therefore, procedural efficiency is a key element to manage resources consumption in hospitals. The aim of this analysis is to assess the hospital efficiency of single-shot cryoballoon ablation (CBA) and irrigated tip point-by-point radiofrequency ablation (RFA), based on the FREEZE Cohort study.

## Methods

### FREEZE Cohort study design

The FREEZE Cohort study (NCT01360008) was a prospective, non-randomized, observational cluster cohort study that compared the effectiveness and safety of RFA and CBA, conducted in 42 experienced hospitals across eight countries [5]. Each centre was assigned as a RFA or a CBA study site, chosen according to the centre’s experience and preference. Patients who received a first ablation for symptomatic paroxysmal or drug-refractory persistent AF were eligible to participate. All enrolled patients were intended to be treated by the designated technique. The study design and the main results of the intention-to-treat analysis were presented in a previous publication [3].

The present analysis evaluates the consequences of differently distributed procedure times between CBA and RFA on schedules and utilisation of resources and staff. Only the procedures undergone in the participating centres across Germany were included as they included all RFA procedures and most of CBA procedures. The calculated metrics and model assumptions were adapted to be illustrative of German hospitals.

### Analysis population

Patients who underwent a PVI-only approach were included in this analysis; PVI procedures with additional ablation lesions (e.g. linear lesions, ablation of complex fractionated atrial electrograms, right atrial flutter, and ablation at the AV node) were excluded. Focal RF touch-ups or treatment with two sizes of the cryoballoon needed in some CBA cases were not excluded.

The treatment groups were defined according to whether CBA or RFA was utilized for the initial ablation, as procedure times should be ascribed to the procedures that were actually completed to reflect clinical reality and to avoid an artificial setting. The crossover rate to any other technique was very low in both groups [3].

### Discrete event simulation model

A discrete event simulation (DES) model was used to evaluate electrophysiology (EP) lab efficiency based on PVI procedure times reported in the FREEZE Cohort. EP lab efficiency for this analysis is defined as improvements in utilization gained from shorter and more predictable procedure times, which includes fewer cumulative overtime hours, more days where overtime is avoided and more days with remaining time for additional EP lab usage.

DES is a method of simulating the behaviour and performance of a real-life process, facility or system, and has been used to model the efficient use of resources in various healthcare settings. These simulations are based on a stochastic time series of individual, granular events representative of realistic occurrences. Individuals and critical resources in the DES are treated distinctly from each other, having unique characteristics and memory, and are drawn from a detailed probabilistic characterization derived from real-world data and experience. SIMUL8 professional version 26.0 (SIMUL8 Corporation) was used for DES in this analysis.

### DES model inputs

**Fig. 1:**
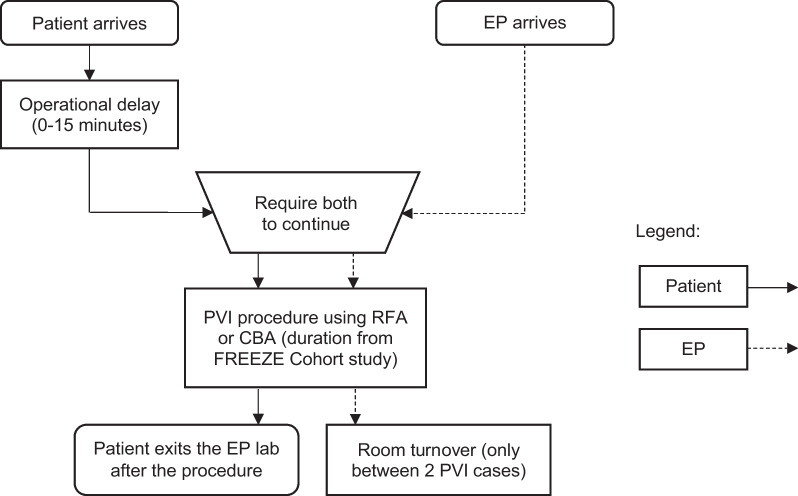
Schematic diagram of the discrete event simulation model. EP, electrophysiologist; CBA, cryoballoon ablation; RFA, irrigated point-by-point radiofrequency ablation; PVI, pulmonary vein isolation. The figure represents a schematic diagram of the discrete event simulation model. On each day, patients arrive at the EP lab for the PVI procedure and experience a one-time delay prior to being available for the case (including a “no delay” time option). When the patient is available, the case will proceed with a randomly selected procedure duration according to the FREEZE Cohort procedure time distribution. Once the procedure is over, the patient leaves the EP lab

This DES aimed to model the impact of operational changes on German EP lab occupancy based on the variability of the ablation procedure start and stop times. In the model, the first PVI case was scheduled at 7:30 am, and three PVI cases were scheduled in a lab day using either the CBA system (Arctic Front™ Cardiac Cryoablation system, Medtronic, Inc.) or an irrigated point-by-point RFA system. The EP lab was modelled fully flexible scheduling: each procedure started as soon as the lab opened or the previous case was over [6]. Operational delays were assumed in the model, including: (1) room turnover time of 20 min and (2) patient delays between 0 and 15 min. No delay associated to the electrophysiologist was assumed in this model. EP lab overtime was counted when PVI procedures extended after 6:00 pm. Figure 1 provides a schematic diagram of the model and Table 1 provides the input values used in the model.

**Table 1:** Discrete event simulation model parameters

Parameter in the DES model	Value
Lab occupancy time distribution*	
CBA procedure time distribution	122.0 ± 39.0 min (FREEZE Cohort)
RFA procedure time distribution	160.0 ± 54.6 min (FREEZE Cohort)
Non-procedural time	38.3 ± 26.5 min (FAST PVI)
Room turnover time	20 min
Lab scheduling method	Fully-flexible scheduling: each procedure starts as soon as the lab opens or as soon as the previous case is over
Dedicated PVI case lab days	3 preplanned cases (PVI)
Shift begin time	7:00 am
First PVI scheduled time	7:30 am
Overtime start time	6:00 pm
Electrophysiologist delay	No delay (dedicated to lab)
Patient operational delay	20–30 min (5% of the time)

CBA, cryoballoon ablation; RFA, irrigated point-by-point radiofrequency ablation; PVI, pulmonary vein isolation

*The time distribution values have been provided in this table with mean ± standard deviation. Distributions of procedure times are available in the Additional file 1

The model used lab occupancy time, defined as the time the patient enters the EP lab until the patient exits it. Lab occupancy time includes both the ablation procedure time (sheath in/out) and non-procedural time during which the patient is in the EP lab, but the procedure is not ongoing. Procedure times were extracted from the FREEZE Cohort study and were separated between CBA and RFA. Non-procedural times were derived from the FAST-PVI study [7] and were similar for both CBA and RFA. Gamma curves were used to fit all procedure time probability distribution data.

### DES model statistics

The length of the base case simulation was set to 1000 lab days to get a procedure time distribution in the model that follows the original one used, from the FREEZE Cohort study. A simulation over 1000 lab days results in an overall average procedure time at ± 1.5% from the FREEZE Cohort mean procedure time, with 95% confidence.

The 1000 lab days simulation was run repeatedly with different random combinations of number seeds, and the final metrics reported represent the overall average of means from these individual runs to ensure the results were not dependent on an arbitrary set of random numbers.

Model outputs were reported as the percent of days leading to overtime, the percent of days with lab time remaining at the end of the planned schedule and the cumulative overtime (hours) over the study period. EP labs have variability in operating parameters such as staff begin and end time, when overtime starts, and room turnover time. For the purposes of a consistent analysis, we have selected parameters generally representative of EP labs and created a simulation model representing efficiency gains in this illustrative EP lab operation.

In addition, sensitivity analyses were performed by individually varying single model inputs (while keeping all other inputs constant), including: (1) lab occupancy time, (2) room turnover time, (3) EP lab shift end, and (4) mean patient delay. Variable values used in the sensitivity analyses are provided in Additional file 1: Table S1. As the DES is stochastic in nature, a separate probabilistic sensitivity analysis was not performed.

### Descriptive statistics

Binary and categorical data are reported as absolute numbers and frequencies, mean and standard deviation or median and quartiles are presented for continuous characteristics of patients treated with CBA and RFA. Procedure time is displayed as a box plot and histogram for both cohorts and the distributions are compared between patient groups by Wilcoxon rank-sum test. The treatment groups are also compared with regard to patient characteristics calculating p-values by Pearson chi-squared test and odds ratios with 95%-confidence intervals for binary variables and using Wilcoxon rank-sum test for ordinal and metrical variables. All tests are two-sided and statistical significance was defined as *p* < 0.05.

The association of patient characteristics with the procedure duration is assessed for CBA and RFA separately by comparing patients above and below a cut-off near the median. Standardized mean differences are calculated showing the direction and magnitude of the imbalance (Austin, Balance diagnostics). The documentation is more than 95% complete with regard to the main variables. No imputation of or adjustment for missing data was performed, with the number of available cases indicated as denominator of rates. Correlation within centre clusters was not taken into consideration. These statistical computations were performed using SAS version 9.4 (Cary, USA).

## Results

### Patient characteristics

In total, 1743 patients enrolled in German hospitals were treated by CBA and 1829 by RFA between April 2011 and February 2016. In CBA group, 138 (7.9%) received PVI plus additional lesions and 382 (20.9%) in RFA group. In RFA group, 67 patients treated with the anatomically-designed PVAC Gold RF catheter were excluded, as this analysis aims to compare point-by-point RFA to CBA. Moreover, 45 (CBA) and 36 (RFA) patients were not available for the analysis because of missing documentation of procedure time. Altogether, 1560 CBA patients and 1344 RFA patients from 30 experienced German centres were included in the analysis.

Relevant patient baseline characteristics are shown in Table 2. The proportion of persistent AF was significantly higher in RFA cohort, and these patients presented with more frequent and more severe symptoms and have more often AF as the current rhythm. Generally, individuals were slightly older, and coronary artery disease, valve disease, and heart failure were more commonly documented as a concomitant disease in this cohort, resulting in a higher risk profile as expressed by the CHA_2_DS_2_-VASc score. On the other hand, CBA patients were more often suffering from hypertensive heart disease.

**Table 2:** FREEZE Cohort patients characteristics

Patient characteristics	CBA	RFA	*P*-value	OR (95% CI)
Number of patients	1560 (53.7%)	1344 (46.3%)		
Age on admission [years]	62.1 ± 10.6, N = 1560	63.2 ± 10.5, N = 1344	0.004	
Female	37.1% (578/1559)	36.2% (486/1342)	0.63	1.04 (0.89–1.21)
Weight [kg]	83 (73, 95)	85 (75, 96)	0.067	
Paroxysmal AF	68.8% (1073/1560)	55.9% (751/1344)	< 0.001	1.74 (1.49–2.03)
Persistent AF (lasting < 1 year)	31.2% (487/1560)	44.1% (593/1344)	< 0.001	0.57 (0.49–0.67)
Frequency of episodes				
Occasionally (less than once per months)	12.2% (183/1503)	5.4% (68/1262)	< 0.001	2.43 (1.82–3.25)
Intermediate (once per month—almost daily)	72.1% (1084/1503)	80.3% (1014/1262)	< 0.001	0.63 (0.53–0.76)
Frequent (at least daily)	15.7% (236/1503)	14.3% (180/1262)	0.29	1.12 (0.91–1.38)
EHRA symptom score III/IV	57.0% (889/1559)	78.3% (1049/1340)	< 0.001	0.37 (0.31–0.43)
Previous non-AF ablation	9.2% (144/1560)	10.1% (136/1344)	0.42	0.90 (0.71–1.16)
Previous device implant*	3.8% (60/1560)	8.0% (107/1344)	< 0.001	0.46 (0.33–0.64)
Number of cadioversions/defibrillation	1.0 ± 1.5, N = 1356	1.2 ± 1.4, N = 901	< 0.001	
Structural heart disease	45.1% (704/1560)	43.3% (580/1341)	0.31	1.08 (0.93–1.25)
Coronary heart disease	10.7% (167/1560)	13.4% (180/1340)	0.024	0.77 (0.62–0.97)
History of myocardial infarction	1.9% (29/1560)	3.6% (48/1340)	0.004	0.51 (0.32–0.81)
Vitium	6.0% (94/1560)	17.6% (236/1340)	< 0.001	0.30 (0.23–0.39)
Aortic valve	1.9% (30/1555)	4.3% (58/1340)	< 0.001	0.43 (0.28–0.68)
Mitral valve	3.5% (55/1555)	11.0% (148/1340)	< 0.001	0.30 (0.21–0.41)
Cardiomyopathy	3.8% (59/1560)	4.3% (57/1340)	0.52	0.88 (0.61–1.28)
Hypertensive heart disease	31.0% (483/1560)	17.2% (231/1340)	< 0.001	2.15 (1.80–2.57)
Primary electrical heart disease	0.8% (12/1560)	2.8% (37/1340)	< 0.001	0.27 (0.14–0.53)
Other leading heart disease	4.4% (63/1428)	2.6% (35/1324)	0.012	1.70 (1.12–2.59)
Heart failure	0.9% (14/1560)	2.5% (33/1343)	< 0.001	0.36 (0.19–0.67)
NYHA functional class III/IV	3.4% (49/1434)	18.3% (195/1068)	< 0.001	0.16 (0.11–0.22)
Cardiac imaging				
LVEF [%]	60 (55, 60)	55 (55, 60)	< 0.001	
Left atrial diameter [mm]	42 (39, 47)	42 (39, 48)	0.027	
Concomitant diseases	70.8% (1105/1560)	78.2% (1050/1343)	< 0.001	0.68 (0.57–0.80)
Hypertension	64.0% (999/1560)	68.1% (914/1343)	0.023	0.84 (0.72–0.98)
Diabetes	6.6% (103/1560)	9.6% (129/1343)	0.003	0.67 (0.51–0.87)
Renal failure (GFR < 60)	4.0% (62/1560)	5.3% (71/1343)	0.092	0.74 (0.52–1.05)
S-Creatinine [mg/dl]	0.9 (0.8, 1.0)	0.9 (0.8, 1.1)	< 0.001	
COPD	2.0% (31/1560)	5.0% (67/1343)	< 0.001	0.39 (0.25–0.59)
Rheumatoid disease	1.0% (16/1560)	2.5% (33/1343)	0.003	0.41 (0.23–0.75)
CHA2DS2-Vasc Score	1.9 ± 1.3, N = 1559	2.1 ± 1.4, N = 1337	< 0.001	
Current rhythm				
Sinus rhythm	86.1% (1343/1560)	66.9% (894/1337)	< 0.001	3.07 (2.55–3.68)
Atrial fibrillation	12.3% (192/1560)	31.4% (420/1337)	< 0.001	0.31 (0.25–0.37)
PM	1.5% (23/1560)	1.0% (14/1337)	0.31	1.41 (0.72–2.76)
Other	0.5% (8/1560)	0.9% (12/1337)	0.21	0.57 (0.23–1.40)

Displayed are percentages and numbers or median and quartiles or mean and standard deviation

*P*-values: Pearson chi-squared test or Mann–Whitney–Wilcoxon test

CBA, cryoballoon ablation; CI, confidence interval, COPD, chronic obstructive pulmonary disease, GFR, glomerular filtration rate, NYHA, New York Heart Association; OR, odds ratio; RFA, point-by-point radiofrequency ablation

*Including pacemakers, implantable cardioverter defibrillators, cardiac resynchronisation therapy devices, cardiac monitors, left atrial appendage occluders

No baseline patient characteristic consistently predicted the duration of RFA or CBA procedure (Additional file 1: Table S2). Therefore, the analysis of the procedure durations was not adjusted for differences in baseline patient characteristics.

### Procedural characteristics

The cryoablation catheter system (Medtronic, Inc.) was used in all CBA cases, 17% from the first generation (Arctic Front), 78% from the second generation (Arctic Front Advance), and 5% from the third generation (Arctic Front Advance ST). The cryoablation protocol (number of freeze cycles, freeze time, use real-time PV potential recordings) was left to the treating physician. In RFA cases, the manufacturers of the RF catheters were Biosense Webster (77.3%), St. Jude (20.8%), Biotronik (1.3%) and others (0.5%). In RFA and CBA groups, 3D electroanatomical mapping was used in 99.5% and 0.1%, respectively (*p* < 0.0001). Intracardiac echocardiography was used in 5.6% and 47.8% of the procedures (*p* < 0.0001), X-ray duration lasted 19 and 17 min (*p* < 0.001) and the dose-area product was 2187 and 1736 cGy*cm^2^ (*p* < 0.001) in average, for RFA and CBA procedures respectively. No information was available on the rate of contact-force-sensing catheters in RFA group [3]. The majority of patients were treated without any sedation or under analgo-sedation in CBA and RFA groups (99.7% vs 99.9% respectively, *p* = 0.53), and only a few patients were treated under endotracheal anaesthesia (0.3% and 0.1% respectively).

**Fig. 2:**
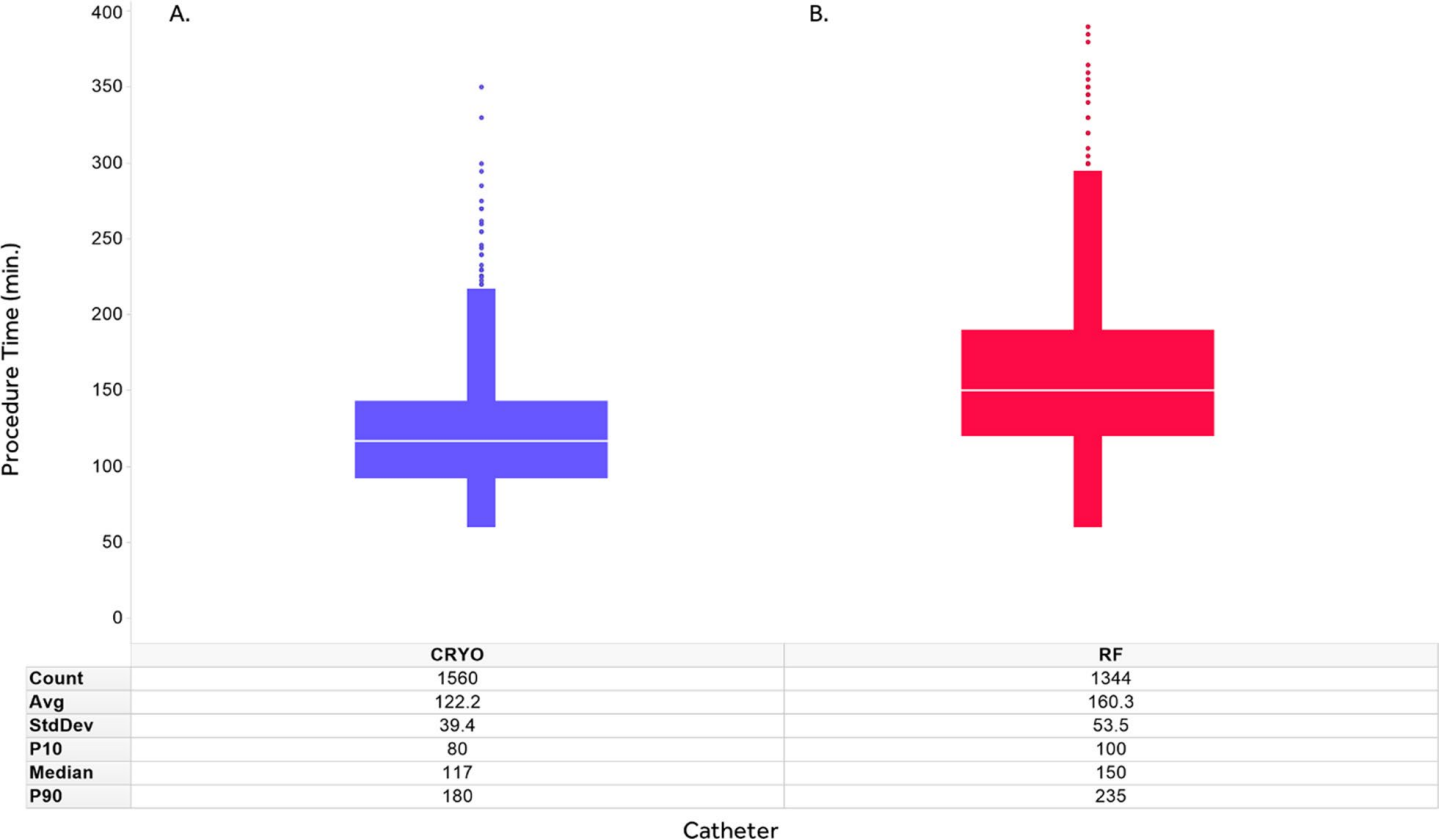
FREEZE Cohort procedure time details. The figure represents the box-plots for CBA and RFA procedure time distributions

**Fig. 3:**
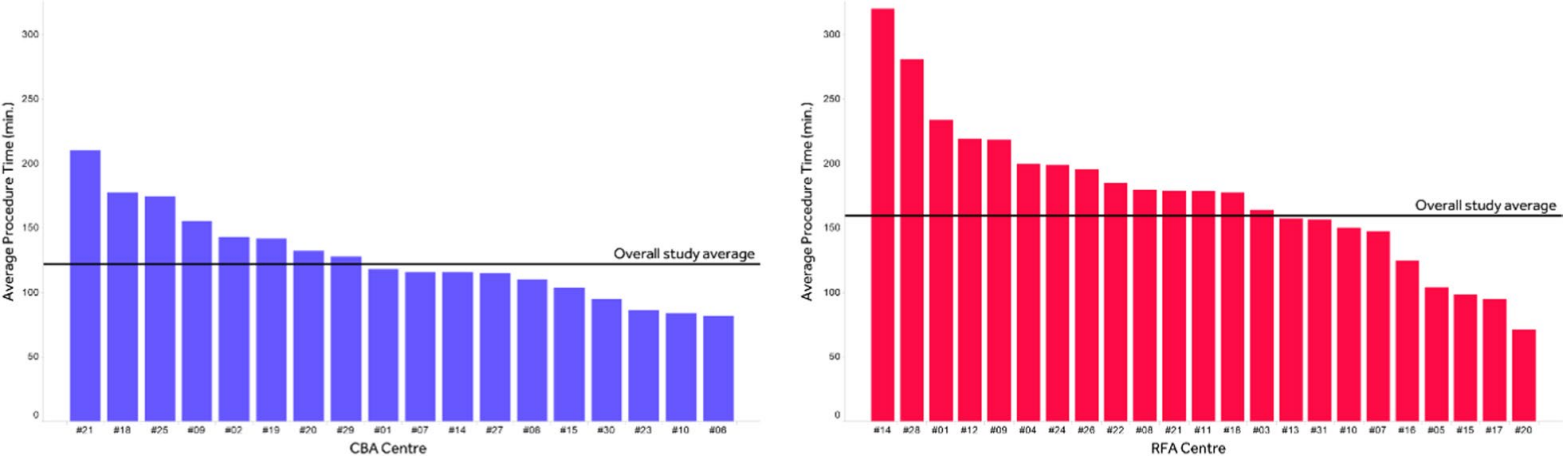
FREEZE Cohort mean procedure times per center. The figure represents the average procedure time for CBA and RFA per center

**Fig. 4:**
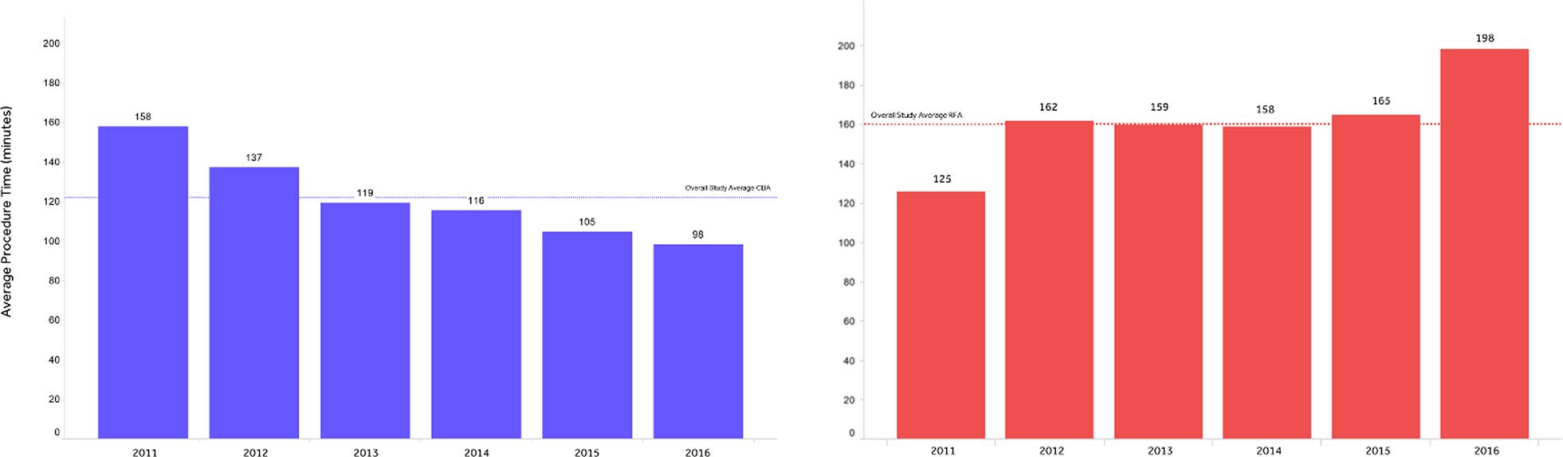
FREEZE Cohort mean procedure times per year. The figure represents the average procedure time for CBA and RFA per year during FREEZE Cohort study period

Figure 2 provides details on the distribution of CBA and RFA procedure durations in the FREEZE Cohort sample. The mean procedure time in CBA cohort was 38 min shorter than in RFA cohort, with smaller standard deviation (122.2 ± 39.4 vs 160.3 ± 53.5 min respectively, *p* < 0.0001). There was also less variability in procedure times between centres undertaking CBA than those with RFA (Fig. 3). Finally, CBA mean procedure time decreased by about an hour over time during the FREEZE Cohort study, whereas RFA mean procedure time seemed fairly steady over the study period (Fig. 4).

### DES model: base case results

**Fig. 5:**
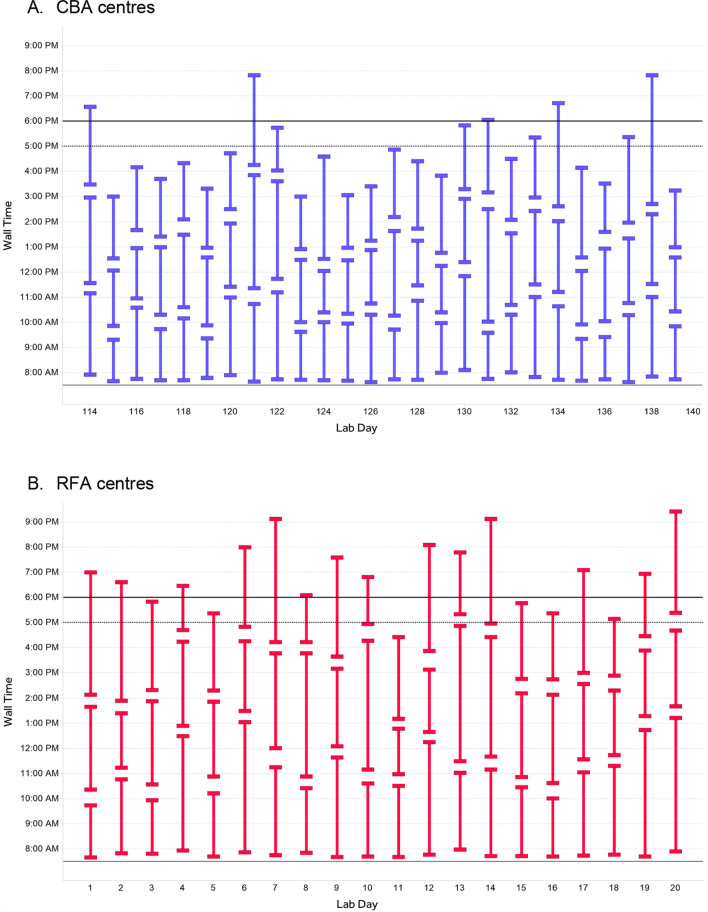
PVI case begin and end times per day: subset simulated lab occupancy. **A** CBA centres and **B** RFA centres. The figure represents the begin and end times for a sampling of days from the simulation, with each contiguous vertical line indicating the time of lab occupancy (the bottom end indicating the case begin time and the top end indicating the case end time) for CBA (**A**) and RFA (**B**) procedures

Results have been consolidated through the repeated run of 1000 lab days simulation for both CBA and RFA. With 3 cases per day simulated for each ablation technique, 3000 PVI using CBA and 3000 using RFA were simulated within the model. A sample of simulated case time distributions is represented in Fig. 5.

**Fig. 6:**
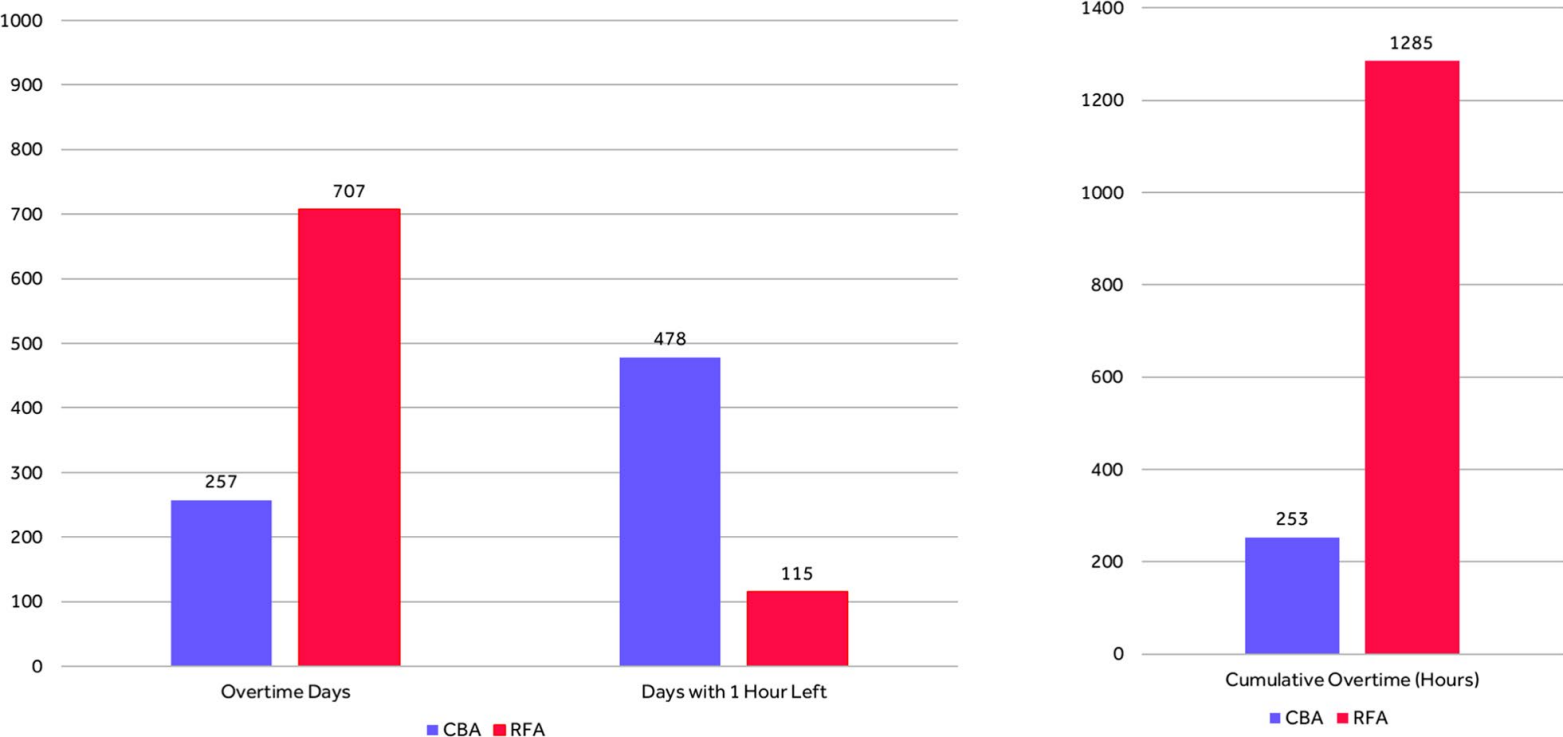
Discrete event simulation model results, 3 metrics after simulation of 1000 lab days. The Fig. 6 represents three DES model metrics after 1000 simulated lab days with PVI using CBA and 1000 using RFA. The metrics are the number of days with overtime, the number of days with an hour left at the end of the EP lab shift and the cumulative overtime in hours

The DES model demonstrated that of the 1000 simulated Cryo lab days, 257 (25.7%) days required overtime to complete the three PVI cases. In contrast, 707 (70.7%) of the simulated RF lab days required overtime to complete the three PVI cases. In total, the cumulative overtime associated with CBA was 253 h during the studied period whereas 1285 h of overtime were required to complete RFA PVI procedures. Overall, RFA was associated with a more than five-fold increase of cumulative overtime compared to CBA over the course of 1000 PVI cases. The model also demonstrated that CBA was associated with 478 days with a remaining hour at the end of the EP lab shift, compared to 115 days with a remaining hour at the end of the lab day when using RFA (47.8% vs 11.5% days with one remaining hour, respectively). These results are represented in Fig. 6.

### DES model: sensitivity analyses results

The sensitivity analyses explored varying the simulation inputs. The results illustrated that the model outputs are most sensitive to procedure time. In all input scenarios, the advantage of CBA as compared to RFA was preserved for all model outputs. All sensitivity analyses and the results are available in the Additional file 1.

## Discussion

Procedure times illustrated in this report from the FREEZE Cohort study demonstrate that PVI using CBA is a significantly shorter, more predictable and more efficient procedure than ablation using RFA. Furthermore, procedure times were more consistent across centres with CBA and so the impact of variability on EP lab efficiency was less pronounced. Also, CBA trend of decreasing procedure time over years during FREEZE Cohort study period was striking, and this characteristic is still observed today, even after many years of CBA use [8]. These findings are consistent with previously published studies [2, 9, 10].

Recent studies have used DES to model AF ablation. Kowalski [11] used lab occupancy times from the VALUE PVI trial to assess the economic impact of procedural efficiencies between CBA and RFA in the United States (US). In this analysis, CBA for paroxysmal AF was associated with a reduction of 36.2% in days with overtime, 92.7% less cumulative overtime hours, and an increase of 46.7% in days with time for an additional EP lab usage. Monnickendam [6] also compared the impact on average procedure consumptions of AF ablation with CBA and RFA via a DES model, using data from a single surgical centre in Belgium. The study demonstrated that 28.6% of RFA procedures are over 30 min longer than the median duration, compared to only 4.4% of CBA procedures. The authors concluded that the choice of AF ablation technology has a significant impact on the operating room efficiency and should be considered in decision-making. Kowalski [12] evaluated EP lab utilization with a DES model using the single-arm STOP Persistent AF trial data in the US, and demonstrated that using CBA to treat persistent AF patients confers EP lab efficiencies that can support an additional third PVI case in a lab day, as opposed to the standard of two in the US. The FREEZE Cohort analysis results are consistent with these previously published DES analyses: hospitals benefit from CBA through efficient use of the EP lab resources, including remaining time for physicians and staff, for the same volume of patients treated. While the extra hours preserved by using CBA instead of RFA are represented here by avoidance of overtime, it may not be overtime avoidance for every centre. However, completing PVI cases more quickly is likely to lead to meaningful advantages, whether it be for avoiding overtime or freeing up lab capacities. With more efficient CBA procedures particularly with streamlined protocols and the latest generation of the cryoballoon [8], there is opportunity for more than 3 PVI procedures to be undergone during an EP lab day. This analysis used German centers procedural data and applied an illustrative EP lab case. The efficiency profile of CBA proved by the present and previously published studies provides a reliable trend to other hospitals with a fully flexible scheduling, from geographies beyond Germany.

Unpredictable and long duration PVI procedures cause overtime which can be associated with an under-utilization of EP lab time and less optimal use of staff resources. Moreover, overtime can be a human resource issue for some hospitals, where the scheduled staff shifts cannot cover the overtime hours, particularly given the low physician and nurse to hospital bed ratios in Germany [13]. The additional hours of overtime with RFA compared to CBA could also represent incremental costs to the hospitals that remunerate overtime hours at a higher tarif than usual. Finally, overtime may contribute to a high turnover of EP lab staff and reduce the efficiency and effectiveness of the workforce in delivering high quality and safe care [14]. In addition to ensuring optimal patient care, employee satisfaction is an important consideration for the employers in a context of shortage of skilled health workers [13].

A previously published analysis of FIRE and ICE trial (NCT01490814) estimated that CBA was associated with meaningful and persistent cost-savings across multiple different healthcare systems, including a total cost-saving of €245,000 during the trial period for German system, due to fewer rehospitalizations, cardioversions and repeat ablations compared to RFA [15, 16]. The FREEZE Cohort study also demonstrated fewer all-cause rehospitalizations and repeat ablations with CBA compared to RFA in real-world settings [3]. Beyond substantial cost-savings for German payers, CBA is also a time-efficient procedure to treat AF patients, with an efficient use of EP lab resources in hospitals in the German healthcare setting.

Given the increasing prevalence of AF, it is important that the clinical community increases access to AF ablation and delivers rhythm control therapies in a timely fashion, particularly, if one considers that today only 5% of all AF patients undergo catheter ablation. The EAST-AFNET trial demonstrated that early rhythm control improves patient outcomes [17]. Moreover, the EARLY-AF [18], STOP AF First [19] and CRYO-First [20] trials illustrate that an early AF ablation with CBA is superior to anti-arrhythmic drug therapy in drug-naïve patients regarding recurrence of atrial tachyarrhythmia after one year. This evidence suggests that the demand for AF ablation is likely to increase even faster than AF prevalence. This additional strain on the healthcare system will reinforce the need to streamline all aspects of AF ablation procedures in order to ensure that patients are treated as efficiently, effectively and safely as possible.

### Limitations

Firstly, the FREEZE Cohort study was not randomized, and patient groups were different at the baseline for some parameters. However, no baseline patient characteristic consistently predicted the duration of RFA or CBA procedure. Also, basing DES analysis on a real-world settings study data provides an added value for simulation results robustness. Secondly, there was no collected data from the FREEZE Cohort study to undertake direct comparisons of the measures predicted by the model: cumulative overtime, days with overtime and days with a remaining hour. Although, DES modelling benefit is to understand impacts that are not easily measured in the real world. This analysis did not address additional measures of EP lab efficiency, such as staffing levels and equipment logistics. The specific gains in efficiency for centres that have different operating parameters than what was chosen for this analysis might deviate, but the sensitivity analyses demonstrate that the gains are likely to be preserved in some form for all reasonable variations of the operating parameters. Thirdly, the FREEZE cohort study did not collect different levels of operators involved in the procedure. Level of experience can be a factor for the duration of the procedure, with less experience resulting in longer procedure times. This effect was addressed in the sensitivity analysis; the impact of reasonable variations in procedure times is explored in the Additional file 1: Fig. S4. The main results of the study remained true even with this variation. Fourthly, only German centres procedural data were included in this analysis to complete the simulation of one representative EP lab case. The vast majority of patients in both groups were treated under analgo-sedation or without any sedation. It remains unclear if the results can be transferred to settings with general anaesthesia. In addition, there are other procedure details that were not explicitly taken into account in the model. However, the variability in procedure times due to these details were reflected in the model, and the efficiency profile of CBA in this model is consistent with previously published studies [2, 9, 10]. Thus, this report results are expected to provide a reliable trend to other hospitals with a fully-flexible scheduling, from geographies beyond Germany. Also, radiation exposure was not included in the DES model, but the associated results from FREEZE Cohort were previously published [3]. Procedure times were collected from the FREEZE Cohort study, and are expected to be lower today because of the current efficiency of AF ablation procedures and based on the advanced ablation technology. The number of PVI per EP lab day, directly linked to the EP lab time per case, is also expected to be higher in most of the centres, however the EP lab efficiency benefit of CBA over RFA is expected to remain. Finally, no information on the rate of contact-force-sensing catheters applied in the RF group, which might potentially influence procedural parameters as well as acute and clinical outcomes, can be provided in our analysis.

## Conclusion

FREEZE Cohort analysis confirms in real-world settings that CBA is faster and more predictable than RFA, and enables improvements in EP lab efficiency. The DES of the FREEZE Cohort data demonstrated the benefits of AF ablation procedures using the cryoballoon system, including fewer cumulative overtime hours, more days where overtime is avoided and more days with remaining time for the staff or for any EP lab usage.

## Supplementary Information

**Additional file 1**.** Figure S1**. Procedure time distribution for Cryoballoon ablation (CBA) procedures (FREEZE Cohort, German CBA centres).** Figure S2**. Procedure time distribution for point-by-point radiofrequency ablation (RFA) procedures (FREEZE Cohort, German RFA centres).** Figure S3**. Plot gamma curves for procedure time distribution (FREEZE Cohort, German centres).** Table S1**. DES model parameters used for the study (Base Case) and the sensitivity analyses.** Figure S4**. Sensitivity analyses results.** Table S2**. Patients baseline characteristics below and above median procedure time for CBA and RFA.

## Data Availability

The data underlying this article cannot be shared publicly due to privacy of the individuals that participated in the study. The datasets analysed during the current study are not publicly available due to privacy of the individuals that participated in the study but are available from the corresponding author on reasonable request.

## Abbreviations

AF: Atrial fibrillation
PVI: Pulmonary vein isolation
CBA: Cryoballoon ablation
RFA: Radiofrequency ablation
DES: Discrete event simulation
EP: Electrophysiology
US: United States

## Code availability

Software SIMUL8 professional version 26.0 (SIMUL8 Corporation).
