# Correction: Electrophysiology lab efficiency comparison between cryoballoon and point-by-point radiofrequency ablation: a German sub-analysis of the FREEZE Cohort study

**DOI:** 10.1186/s12872-023-03107-z

**Published:** 2023-03-01

**Authors:** Andreas Metzner, Florian Straube, Roland R. Tilz, Malte Kuniss, Georg Noelker, Juergen Tebbenjohanns, Dietrich Andresen, Heinrich Wieneke, Christoph Stellbrink, Jennifer Franke, Uwe Dorwarth, Phuong Lien Carion, Reece Holbrook, Matthias Hochadel, Jochen Senges, Ellen Hoffmann, Karl-Heinz Kuck, for the FREEZE Cohort Study Investigators, L. Q. Wu, A. Garcia-Alberola, T. Massa, G. Sabin, A. Franke, J. J. Souza, A. Stanley, S. G. Spitzer, S. Willems, T. Dierk, K. R. J. Chun, R. Borchard, K. H. Seidl, R. Zahn, G. Groschup, I. W. P. Obel, J. Brachmann, J. H. Gerds-Li, R. R. Gopal, J. Schrickel, T. Lewalter, A. Stanley, W. Moshage, L. Eckardt, W. Jung, P. Kremer, A. Lubinski, B. Schumacher, L. Lickfett, T. Münzel, C. Steinwender, M. Efremidis, T. Deneke, D. Q. Nguyen

**Affiliations:** 1 Department of Cardiology, Asklepios Klinik St. Georg, Hamburg, Germany; 2 Department of Cardiology, Universitätsklinikum Hamburg-Eppendorf, Martinistraße 52, Gebäude Ost 70, 20246 Hamburg, Germany; 3 Department of Cardiology and Internal Intensive Care Medicine, Heart Center Munich-Bogenhausen - Munich Municipal Hospital Group, Munich, Germany; 4 Department of Cardiology, Angiology, and Intensive Care Medicine, University Hospital Schleswig-Holstein, University Heart Centre Luebeck, Lübeck, Germany; 5 Department of Cardiology, Kerckhoff-Klinik, Bad Nauheim, Germany; 6 Herz- Und Diabeteszentrum Nordrhein-Westfalen, Bad Oeynhausen, Germany; 7 HELIOS Klinikum Hildesheim, Medizinische Klinik I – Kardiologie, Hildesheim, Germany; 8 Department of Cardiology Paul Gerhardt Diakonie gAG, Evangelisches Krankenhaus Hubertus, Berlin, Germany; 9 Klinik Für Kardiologie Und Angiologie, Contilia Herz- Und Gefäßzentrum, Essen, Germany; 10 Department of Cardiology, Klinikum Bielefeld, Bielefeld, Germany; 11 CardioVascular Center Frankfurt, Frankfurt, Germany; 12 Medtronic International Trading Sàrl, Tolochenaz, Switzerland; 13 Medtronic, Inc, Mounds View, MN USA; 14 Stiftung Institut Fur Herzinfarktforschung, Ludwigshafen, Germany

**Correction to: BMC Cardiovascular Disorders (2023) 23:8** https://doi.org/10.1186/s12872-022-03015-8

Following publication of the original article, it came to the author's attention that Prof. Andrzej Lubinski’s affiliation (in the ‘FREEZE Cohort Study Investigators’ section) affiliation was incorrect. The article has since been updated with the corrected affiliation, which is as follows: Medical University of Gdańsk, Poland.

