# Supplementary material for: Electrophysiology lab efficiency comparison between cryoballoon and point-by-point radiofrequency ablation: a German sub-analysis of the FREEZE Cohort study
Source: BMC Cardiovasc Disord. 2023 Jan 9;23:8. doi: 10.1186/s12872-022-03015-8 (PMC9830778; doi:10.1186/s12872-022-03015-8)
Supplement: Supplementary file 1 — Additional file 1. Figure S1. Procedure time distribution for Cryoballoon ablation (CBA) procedures (FREEZE Cohort, German CBA centres). Figure S2. Procedure time distribution for point-by-point radiofrequency ablation (RFA) procedures (FREEZE Cohort, German RFA centres). Figure S3. Plot gamma curves for procedure time distribution (FREEZE Cohort, German centres). Table S1. DES model parameters used for the study (Base Case) and the sensitivity analyses. Figure S4. Sensitivity analyses results. Table S2. Patients baseline characteristics below and above median procedure time for CBA and RFA. [file 12872_2022_3015_MOESM1_ESM.docx]

**ADDITIONAL FILE 1**

**Electrophysiology Lab Efficiency Comparison Between Cryoballoon and Point-by-Point Radiofrequency ablation: a German Sub-Analysis of the FREEZE Cohort Study**

**Definitions**

**Lab occupancy time**: time from the patient is wheeled in the EP lab until the patient is wheeled out, corresponding to the sum of the procedure time and the non-procedural time.

**Procedure time**: total time from a sheath is inserted in the patient until it is out for the entire ablation procedure (FREEZE Cohort study^[1]^).

**Non-procedural time**: time during which the patient is in the EP lab, before and after the ablation procedure is undertaken by the electrophysiologist (FAST PVI study^[2]^).

**Room turnover time**: time between two procedures, when there is no patient in the EP lab.

**Lab occupancy time: distribution**

**Additional file 1: Figure S1**: Procedure time distribution for Cryoballoon ablation (CBA) procedures (FREEZE Cohort, German CBA centres).

**Additional file 1:** **Figure S2**: Procedure time distribution for point-by-point radiofrequency ablation (RFA) procedures (FREEZE Cohort, German RFA centres).

**Additional file 1:** **Figure S3**: Plot gamma curves for procedure time distribution (FREEZE Cohort, German centres).

**Sensitivity analyses**

**Additional file 1:** **Table S1**: DES model parameters used for the study (Base Case) and the sensitivity analyses.

| Parameter | Base Case Value | Sensitivity analysis:  Low Value | Sensitivity analysis:  High Value |
| --- | --- | --- | --- |
| **Lab Occupancy Time Distribution** | Procedure times used are based on FREEZE Cohort study, with procedures from all German centres | Procedure times used are based on German centres from the FREEZE Cohort study that had a mean procedure time faster than the average procedure time from all the German centres | Procedure times used are based on German centres from the FREEZE Cohort study that had a mean procedure slower than the average procedure time from all German centres |
| **Overtime Start** | 6:00pm | 5:30pm | 6:30pm |
| **Room Turnover Time** | 20 minutes | 15 minutes | 45 minutes |
| **Patient Operational Delay** | 20-30 minutes  (5% of the time) | 20-30 minutes  (2% of the time) | 20-30 minutes  (10% of the time) |

The “High Value” is the high-end of the range for the variables, and the “Low Value” is numerically lower than the base case value based on ranges for each parameter.

**Additional file 1:** **Figure S4**: Sensitivity analyses results.

Figure S4 is composed of six tornado charts, showing the impact of varying the model inputs according to the High and Low values from Table S1.

A & D: The horizontal axis represents the number of overtime days according to the DES model. The limits of the horizontal bar associated with each variable indicate the number of overtime days when the indicated parameter is changed to the low value (light blue for CBA and pink for RFA) or high value (dark blue for CBA and red for RFA) while holding the other variables constant.

B & E: The charts represent the number of days with an hour left within the model when each variable is changed independently to the low (light blue for CBA and pink for RFA) or high (dark blue for CBA and red for RFA) value.

C & F: The charts represent the number of cumulative overtime hours within the model when each variable is changed independently to the low (light blue for CBA and pink for RFA) or high (dark blue for CBA and red for RFA) value.

**Patients baseline characteristics analyses**

**Additional file 1:** **Table S2**: Patients baseline characteristics below and above median procedure time for CBA and RFA.

|  |  | **CBA** |  |  | **RFA** |  |
| --- | --- | --- | --- | --- | --- | --- |
|  | **> 120 min** | **≤ 120 min** | **SMD** | **> 150 min** | **≤ 150 min** | **SMD** |
|  | | | |  |  |  |
| **Number of patients** | 650 | 910 |  | 637 | 707 |  |
| Age on admission [years] | 62.2 ± 10.8 | 62.0 ± 10.4 | +0.03 | 62.1 ± 10.9 | 64.1 ± 10.1 | -0.19 |
| Female | 35.4 % | 38.2 % | -0.06 | 33.9 % | 38.3 % | -0.09 |
| Weight [kg] | 86.0 ± 17.3 | 84.8 ± 17.0 | +0.07 | 88.6 ± 18.5 | 85.1 ± 18.0 | +0.19 |
|  |  |  |  |  |  |  |
| Paroxysmal AF | 63.1 % | 72.9 % | -0.21 | 55.4 % | 56.3 % | -0.02 |
| Frequent episodes | 12.1 % | 18.4 % | -0.18 | 19.9 % | 9.4 % | +0.30 |
| EHRA symptom score III/IV | 59.5 % | 55.2 % | +0.09 | 74.6 % | 81.7 % | -0.17 |
| Private health insurance | 36.9 % | 25.8 % | +0.24 | 15.4 % | 31.0 % | -0.38 |
|  |  |  |  |  |  |  |
| **Structural heart disease** | 50.3 % | 41.4 % | +0.18 | 45.2 % | 41.5 % | +0.08 |
| Coronary heart disease | 10.9 % | 10.5 % | +0.01 | 15.1 % | 11.9 % | +0.09 |
| Vitium | 6.9 % | 5.4 % | +0.06 | 15.3 % | 19.7 % | -0.12 |
| Cardiomyopathy | 4.8 % | 3.1 % | +0.09 | 4.2 % | 4.3 % | -0.00 |
| Hypertensive heart disease | 37.8 % | 26.0 % | +0.26 | 20.4 % | 14.3 % | +0.16 |
| Primary electrical heart disease | 0.3 % | 1.1 % | -0.09 | 3.3 % | 2.3 % | +0.06 |
| Other leading heart disease | 3.1 % | 5.2 % | -0.10 | 2.5 % | 2.8 % | -0.02 |
| Heart failure | 0.8 % | 1.0 % | -0.02 | 3.6 % | 1.4 % | +0.14 |
|  |  |  |  |  |  |  |
| NYHA functional class III/IV | 3.3 % | 3.5 % | -0.01 | 12.7 % | 23.8 % | -0.29 |
|  |  |  |  |  |  |  |
| **Cardiac Imaging** |  |  |  |  |  |  |
| LVEF [%] | 57.6 ± 5.7 | 58.7 ± 7.1 | -0.17 | 57.7 ± 7.2 | 56.6 ± 6.4 | +0.16 |
| LA Diameter [mm] | 44.1 ± 7.0 | 42.2 ± 6.5 | +0.28 | 43.5 ± 6.9 | 43.5 ± 6.3 | -0.00 |
|  |  |  |  |  |  |  |
| **Concomitant diseases** | 75.4 % | 67.6 % | +0.17 | 79.3 % | 77.2 % | +0.05 |
| Hypertension | 69.7 % | 60.0 % | +0.20 | 69.7 % | 66.6 % | +0.07 |
| Diabetes | 6.3 % | 6.8 % | -0.02 | 11.1 % | 8.2 % | +0.10 |
| Renal failure (GFR < 60) | 4.5 % | 3.6 % | +0.04 | 6.4 % | 4.2 % | +0.10 |
| S-Creatinine [mg/dl] | 1.0 ± 0.2 | 0.9 ± 0.2 | +0.15 | 1.0 ± 0.3 | 1.0 ± 0.3 | +0.08 |
| COPD | 1.7 % | 2.2 % | -0.04 | 6.4 % | 3.7 % | +0.13 |
| Rheumatoid disease | 0.8 % | 1.2 % | -0.04 | 3.1 % | 1.8 % | +0.08 |
| CHA2DS2-Vasc Score | 1.9 ± 1.3 | 1.8 ± 1.3 | +0.09 | 2.1 ± 1.5 | 2.1 ± 1.3 | +0.02 |
|  |  |  |  |  |  |  |
| Current AF | 13.5 % | 11.4 % | +0.06 | 28.1 % | 34.4 % | -0.14 |
|  |  |  |  |  |  |  |
| Displayed are percentages and numbers or mean and standard deviation SMD: Standardized difference | | | | | | |

1. Hoffmann E et al. Outcomes of cryoballoon or radiofrequency ablation in symptomatic paroxysmal or persistent atrial fibrillation. Europace 2019;21:1313-1324.
2. Klein G et al. Comparison of ‘anatomically designed’ and ‘point-by-point’ catheter ablations for human atrial fibrillation in terms of procedure timing and costs in German hospitals. Europace 2015; 17(7):1030–1037.
